# Vaccinia virus BTB-Kelch proteins C2 and F3 inhibit NF-κB activation

**DOI:** 10.1099/jgv.0.001786

**Published:** 2022-10-01

**Authors:** Rui-Yao Zhang, Mitchell A. Pallett, Jamie French, Hongwei Ren, Geoffrey L. Smith

**Affiliations:** Department of Pathology, University of Cambridge, Tennis Court Road, Cambridge CB2 1QP, UK

**Keywords:** BTB-Kelch, CD8+ T cell, immune evasion, NF-κB signalling, vaccination, vaccinia virus

## Abstract

Vaccinia virus (VACV) encodes scores of proteins that suppress host innate immunity and many of these target intracellular signalling pathways leading to activation of inflammation. The transcription factor NF-κB plays a critical role in the host response to infection and is targeted by many viruses, including VACV that encodes 12 NF-κB inhibitors that interfere at different stages in this signalling pathway. Here we report that VACV proteins C2 and F3 are additional inhibitors of this pathway. C2 and F3 are BTB-Kelch proteins that are expressed early during infection, are non-essential for virus replication, but affect the outcome of infection *in vivo*. Using reporter gene assays, RT-qPCR analyses of endogenous gene expression, and ELISA, these BTB-Kelch proteins are shown here to diminish NF-κB activation by reducing translocation of p65 into the nucleus. C2 and F3 are the 13^th^ and 14^th^ NF-κB inhibitors encoded by VACV. Remarkably, in every case tested, these individual proteins affect virulence *in vivo* and therefore have non-redundant functions. Lastly, immunisation with a VACV strain lacking C2 induced a stronger CD8+ T cell response and better protection against virus challenge.

## Introduction

*Vaccinia virus* (VACV) is a member of the *Orthopoxvirus* genus of the *Poxviridae* [1]. It is famous as the live vaccine used to eradicate smallpox, an achievement certified by the World Health Organisation in 1979 [2]. Despite the eradication of smallpox, interest in VACV has continued because of its development as vaccines against other infectious diseases, as an oncolytic agent and as an excellent model to study host-pathogen interactions.

The VACV genome is a linear double-stranded DNA molecule of about 191 kbp [3] and can be divided into a conserved central region of about 100 kb and more variable terminal regions [4]. The central region encodes proteins that are needed for virus replication, whilst the terminal regions encode proteins that affect host range, virulence and immune evasion. VACV encodes scores of proteins that inhibit the innate immune response to infection [5] and the study of these proteins is enhancing understanding of not only host-pathogen interactions, but also of the immune system. For instance, specific VACV proteins were found to control body temperature of the infected mammal [6], induce a hypoxic response during normoxia [7], or induce proteasomal degradation of histone deacetylase 4 (HDAC4), a newly identified component of the type I interferon (IFN) signalling pathway [8]. Many of the VACV immunomodulatory proteins target intracellular signalling pathways leading to the activation of the transcription factors IRF3 or NF-κB, or the expression of IFN-stimulated genes (ISGs). For reviews of these immunomodulatory proteins see [5, 9].

NF-κB is a critical transcription factor needed for the production of IFN and the inflammatory response to infection. Until recently, 11 VACV proteins have been described that inhibit NF-κB activation within the infected cell. These are A46 [10], A49 [11, 12], A52 [10], A55 [13], B14 [14], C4 [15], E3 [16], K1 [17, 18], K7 [19], M2 [20, 21] and N1 [22–24]. In addition, VACV encodes proteins that are secreted from the infected cell to prevent IL-1β [25, 26] or TNFα [27, 28] binding to cognate receptors and so prevent NF-κB signalling. Despite this formidable array of inhibitors, a VACV strain engineered to lack these intracellular proteins still blocked NF-κB activation [29], indicating the presence of additional inhibitor(s). Recently, protein F14 was identified as a 12^th^ inhibitor that functions within the nucleus by molecular mimicry of the transactivation domain of p65/RelA [30].

VACV strain Western Reserve (WR) encodes three proteins A55, C2 and F3 that are related to the cellular BTB (Bric-a-brac, Tramtrack and Broad-complex)-Kelch protein family. These are intracellular polypeptides that are expressed early during infection and, although non-essential for virus replication in cultured cells, they affect the cytopathic effect induced by VACV infection of cultured cells, and the host response to infection *in vivo* [31–33]. BTB-Kelch proteins are substrate adaptors for the cullin-3 (Cul3)-RING based E3 ubiquitin ligase complex and are involved in a wide variety of biological processes such as transcriptional regulation, cytoskeletal arrangement, ion conductance and protein ubiquitylation [34–36]. The N-terminal BTB-BACK domains engage Cul3, and the C-terminal Kelch domain recognises specific substrates [37–39] that can then be ubiquitylated to modify their function or induce their proteasomal degradation. For example, human BTB-Kelch proteins KLHL13 and KLHL9 bind to Cul3, but not to Cul1, Cul2, Cul4A or Cul5, indicating their selective interaction [40].

BTB-Kelch proteins are encoded by several orthopoxviruses, for review see [41]. For example, ectromelia virus (ECTV) strain Moscow proteins EVM150 and EVM167 co-precipitate with Cul3 [42] and EVM150 inhibits NF-κB activation [43]. VACV protein A55, a closely related orthologue of ECTV EVM150, was shown to interact with Cul3 and inhibit NF-κB in a Cul3-independent manner [13]. The crystal structure of the A55 BTB-BACK region in complex with the N-terminal domain of Cul3 was determined and A55 had a very high affinity for Cul3 and greater than the affinity of cellular BTB-Kelch proteins for Cul3 [44].

Here, VACV BTB-Kelch proteins C2 and F3 are described as additional inhibitors of NF-κB that function to diminish translocation of p65 into the nucleus in a Cul3-independent way. Further analysis of a VACV strain lacking C2, vΔC2, showed that it induced a stronger VACV-specific CD8+ T cell memory response following vaccination of mice.

## Methods

### Cells, plasmids, reagents and viruses

BSC-1, HEK-293T, HeLa and RK-13 cells were maintained in Dulbecco’s modified Eagle’s medium (DMEM) with high glucose (Gibco) and supplemented with 10 % (v/v) foetal bovine serum (FBS; Pan Biotech), 1 % penicillin/streptomycin (P/S) and 1 % MEM non-essential amino acids (NEAA; Gibco) at 37 °C in a 5 % CO_2_ atmosphere. All plasmids used and those constructed during this study are listed in Table S1 (available with the online version of this article). Plasmids were generated using conventional restriction enzyme (RE) digestion and ligation using the primers and RE sites listed in Table S1. All reagents were purchased from Sigma unless stated otherwise. A plaque-purified wild-type vaccinia virus (VACV) strain Western Reserve (WR) (vC2) and derivative mutant lacking gene *C2L* (vΔC2), and revertant virus containing *C2L* that had been reinserted into vΔC2 at its natural locus (vC2-Rev) were described [31]. Virus infectivity was titrated by plaque assay on BSC-1 cells.

### Generation of HEK-293T pLDT cell lines expressing BTB-Kelch proteins

pLDT cells lines that inducibly express C2, F3 or their BTB-BACK or Kelch domains with a N-terminal tandem affinity purification (TAP)-tag, consisting of two Streptavidin binding sequences and one Flag tag [45], or empty vector (EV) were constructed using the pLKO-based lentivirus vector plasmids listed in Table S1. C2-B, C2-BTB-BACK (aa 1-212): C2-K, C2-Kelch (aa 213−512): F3-B, F3-BTB-BACK (aa 1−263): F3-K, F3-Kelch (aa 264−480). The plasmids were co-transfected with the envelope plasmid pVSV-G and a helper plasmid pCMV-dR8.91 for lentivirus production in HEK-293T using the LT1 transfection reagent. After 48 h the medium was filtered and transferred to fresh monolayers of HEK-293T and 3 d later 2 μg ml^−1^ puromycin (Invivogen) was added. Transduction and expression was confirmed by the addition of 2 μg ml^−1^ doxycycline and immunoblotting of cell lysates.

### Luciferase reporter assay

HEK-293T and HeLa cells were seeded in 96-well plates and when 70−80 % confluent were transfected with either the NF-KB-Luciferase reporter plasmid (pNF-KB-Luc) or the IFN-stimulated response element-Luciferase reporter plasmid (pISRE-Luc) and a plasmid constitutively expressing Renilla Luciferase (pTK-RL). Following stimulation, cells were lysed in passive lysis buffer (Promega) and Firefly luciferase and Renilla luciferase were measured with Firefly luciferase substrate and Renilla luciferase substrate (Nanolight Technology) using a FLUOstar luminometer (BMG). The Firefly Luciferase activity was normalised to the Renilla Luciferase activity first, and then data were normalised to non-stimulated EV group. At least three independent (technical replicates) measurements were taken per condition per experiment.

### RT-qPCR

RNA extraction, cDNA synthesis and RT-qPCR were carried out as described [46]. qPCR used primers for *IL-8* (AGAAACCACCGGAAGGAACCATCT and AGAGCTGCAGAAATCAGGAAGGCT) and *GAPDH* (TCGACAGTCAGCCGCATCTTCTTT and ACCAAATCCGTTGACTCCGACCTT).

### ELISAS

HEK-293T pLDT cell lines were starved for 4 h in DMEM without supplements before stimulating for 18 h with 40 ng ml^−1^ TNFα. IL-8 in the medium was measured using human IL-8/CXCL8 DuoSet ELISA kit (R and D Systems) and the FLUOstar Omega Luminometer (BMG Labtech). Experiments were carried out in triplicate and measured with technical repeats.

### p65 translocation assay and immunofluorescent staining

HeLa cells were transfected with EV or plasmids encoding N-terminal TAP-tagged B14, C2, C2-B (aa 1−212), C2-K (aa 213−512) or F3 using the LT1 transfection reagent (MirusBio). After 24 h cells were starved in DMEM without serum for 3 h and then stimulated with 40 ng ml^−1^ TNFα for 30 min. Cells were washed three times in ice-cold PBS and processed for immunofluorescence staining and imaging as described [47]. Polyclonal rabbit anti-Flag (F7425, Sigma-Aldrich) and mouse monoclonal anti-p65 clone F-6 (sc-8008; Santa Cruz) were used as the primary antibodies and goat anti-rabbit 546 and donkey anti-mouse 488 were used as the secondary antibodies (Jackson Immunoresearch). Images were analysed using the Zeiss Zen microscope software and ImageJ. Experiments were performed in triplicate and carried out three times. For each repeat, 100 Flag-positive cells were analysed for each condition and the percentage of cells showing nuclear p65 determined.

### Immunoprecipitation and immunoblotting

HEK-293T cells were lysed in 1 % NP-40/PBS or RIPA buffer (50 mM Tris pH 8.0, 150 mM NaCl, 0.5 M EDTA, 1 % NP40 [IGEPAL CA-630], 0.5 % sodium deoxycholate, 0.1 % SDS supplemented with protease inhibitor). The lysates were clarified by centrifugation at 15200 ***g*** for 20 min at 4 °C and a sample of the supernatant was retained as whole cell lysate (WCL). The remainder was incubated with anti-FLAG M2 affinity gel (Sigma-Aldrich) at 4 °C overnight, washed in lysis buffer three times, re-suspended in 4× Laemmli buffer and analysed by SDS-polyacrylamide gel electrophoresis (PAGE) in Tris-glycine-SDS (TGS) buffer (20 mM Tris, 192 mM glycine, 1 % (w/v) SDS) followed by immunoblotting. Protein samples were transferred to a nitrocellulose membrane (GE Healthcare) in Tris-glycine (TG) buffer (20 mM Tris-HCl pH 8.3, 150 mM glycine) using the Turboblot system (BioRAD). Membranes were blocked in 5 % milk in Tris-buffered saline (10 mM Tris, 150 mM NaCl) pH 7.4 with 0.1 % (v/v) Tween-20 (TBS-T) for 60 min before incubating with the primary antibody overnight at 4 °C. Primary antibodies used were: rabbit polyclonal anti-IκBα (Cell Signalling Technology [CST], #9242), anti-p65 S536 (CST, S3010S), rabbit monoclonal anti-cullin-3 clone EPR3195 (Abcam, ab108407), anti-Flag (Sigma-Aldrich, F7425), anti-V5 (Bio-Rad; MCA1360GA) mouse monoclonal anti-myc clone 9B11 (CST, #2276), anti-α-tubulin clone DM1A (Millipore; 05−829) and anti-phospho-IκBα (CST, #9246). Membranes were washed three times in TBS-T before incubating with secondary antibodies for 1.5 h at room temperature. Secondary antibodies used were goat anti-rabbit IRDye 800CW (926−68032 211; LiCOR) and goat anti-mouse IRDye 608LT (926−68020; LiCOR). Finally, membranes were washed three times in TBS-T, dried and imaged using the LiCOR system and Odyssey software. Band intensity was calculated using Odyssey.

### *In vivo* work

Viruses used for *in vivo* work were purified by centrifugation through sucrose density gradients. Groups of five C57BL/6 female mice 6−8 weeks old (Envigo) were infected intradermally in the ear pinnae with 1×10^4^ p.f.u. of VACV strains vC2, vΔC2 or vC2-Rev [31]. Lesions were monitored daily as described [48]. For challenge experiments, mice immunised as above 28 d previously were infected intranasally with 1x10^7^ p.f.u. of wild-type VACV WR and the weight change and signs of illness were measured daily thereafter as described [25].

### Flow cytometry

Cells present in infected ears and draining lymph nodes were extracted at 7 d post-infection (p.i.) and analysed by flow cytometry as described [49]. Splenocytes and lymph node suspension cells were obtained by forcing the organ through a stainless steel mesh. Splenocytes were treated with red blood cell (RBC) lysis buffer to remove contaminating RBCs. Single cell suspensions were stained with fluorescence-labelled antibodies: anti-CD3 (clone 145−2 C11), CD4 (GK1.5), CD8 (5H10-1), CD45R (RA-6B2), NK1.1 (PK136), CD69 (H1.2F3), Ly6G (1A8), F4/80 (BM8) and CD16/32 (2.4G2) antibodies were purchased from BD Biosciences or from Biolegend. These antibodies were purified or conjugated with PerCP/cy5.5, FITC, APC/Cy7, APC, PE-Cy7, PE, or BV650. Relevant isotypes were used as control. Flow cytometry was performed with a BD LSR Fortessa (BD Biosciences) and data were analysed with FLOWJO software (Tree Star Inc., Ashland, OR). LIVE/DEAD Fixable Aqua Dead Cell Stain Kit (Life Technologies, Paisley, UK) was used to exclude non-viable cells from analysis.

### DimerX assay to detect VACV-specific CD8^+^ T cells

VACV-specific, splenic, CD8^+^ T-cell responses were measured at 7 and 28 d p.i. as described using the DimerX assay according to the manufacturer’s instructions (BD Biosciences) using H-2K^b^:Ig fusion proteins and B8_20_ peptide (TSYKFESV) [13].

### Statistics

All experiments were carried out in triplicate and are representative of an average of at least three independent biological repeats unless stated. Data are the mean+/-SD, or, for *in vivo* data where stated, +/-SEM. All assays were analysed by unpaired T-test with GraphPad Prism 6 Software where *P* <0.05=*, *P* <0.01=**, *P* <0.001=*** and *P* <0.0001=****.

### Data deposit

Primary data used in the preparation of figures in this manuscript have been deposited at Figshare 10.6084/m9.figshare.20372202.

## Results

### C2 and F3 inhibit activation of the NF-κB signalling pathway

**Fig. 1:**
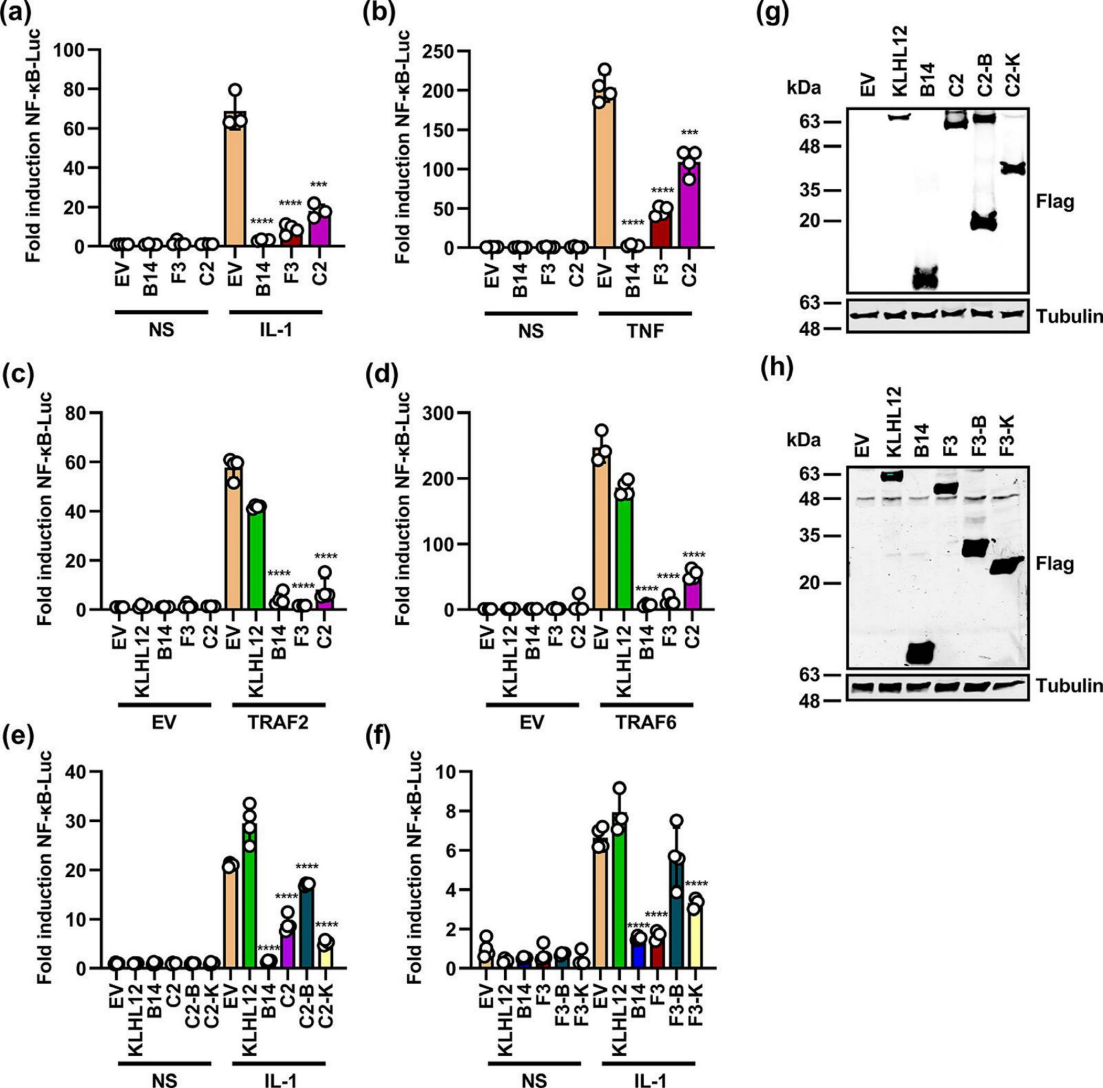
C2 and F3 inhibit activation of NF-κB signalling. (a-d) HEK-293T cells were co-transfected with pNF-KB-Luc, pTK-RL and plasmids for expression of the indicated TAP-tagged proteins. Eighteen hours post-transfection cells were untreated or stimulated by addition of (a) IL-1*β* (20 ng ml^−1^) or (b) TNFα (40 ng ml^−1^) for 6 h or co-transfected with plasmids expressing (c) TRAF2 (40 ng), or (d) TRAF6 (10 ng) for 24 h. Luciferase activity was measured in cell lysates and data are expressed as fold change in Firefly luciferase relative to Renilla and EV unstimulated. (e, f) HEK-293T cells were co-transfected with the pNF-KB-Luc, pTK-RL and plasmids for expression of the indicated TAP-tagged proteins (C2-B, C2-BTB-BACK aa 1−212; C2-K, C2-Kelch aa 213−512; F3-B, F3-BTB-BACK aa 1−263; F3-K, F3-Kelch aa 264−480) and 18 h later were stimulated with IL-1*β*, as above. (g, h) Samples were subjected to SDS-PAGE and immunoblotting and show representative relative protein expression from each plasmid. Molecular masses (in kDa) are indicated on the left. Data shown (mean±SD) are representative of three experiments and were analysed by an unpaired Student’s t-test (**P <0.01, ***P <0.001, ****P <0.0001) in comparison to stimulated EV.

**Fig. 2:**
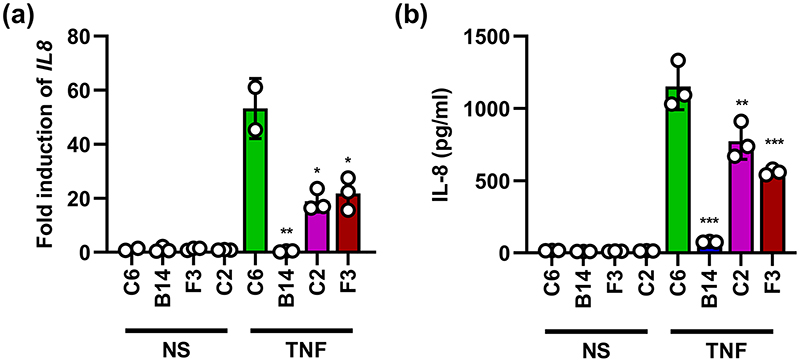
C2 and F3 inhibit NF-κB-dependent transcription and secretion of IL-8. (a, b) HEK-293T cell lines were treated with 2 μg ml ^1^ doxycyctine to induce expression of C6, B14, C2 or F3 for 24 h and then were either left unstimutated or were stimulated with 40 ng mt^−1^ TNFα for 1.5 h. (a) *IL-8* mRNA tevets were analysed by RT-qPCR relative to *GAPDH,* as a house keeping gene. (b) IL-8 ELISA. After doxycyctine treatment, cells were starved for 3 h in DMEM and left unstimutated or stimutated with TNFα for 18 h. Levels of IL-8 in the cell culture medium were assayed by ELISA. Data shown (mean±SD) were performed at teast in triplicate per experiment and are representative of three experiments and were anatysed by an unpaired Student’s t-test (**P <0.01, ***P <0.001, ****P <0.0001) in comparison to C6.

C2 and F3 affect the outcome of VACV infection in an intradermal mouse model [31, 33] but the mechanisms by which they do so remain unclear. Given that the related VACV protein A55 [13] and the corresponding protein from ECTV (EMV150) [43] each inhibit NF-κB activation, the effect of C2 and F3 on NF-κB signalling pathway was examined. HEK-293T cells were co-transfected with plasmids expressing Flag-tagged C2, or F3, and plasmids encoding Firefly luciferase under an NF-κB responsive promoter or Renilla luciferase. Empty vector (EV) was included as a negative control, and VACV protein B14 was included as a known inhibitor of NF-κB signalling [14]. Cells were untreated or stimulated by addition of IL-1β (**a**), or TNFα (**b**) or by transfection with plasmids expressing TRAF2 (c) or TRAF6 (**d**) and the luciferase activity was measured in cell lysates. To ensure that any inhibitory effects observed upon overexpression of TRAF2 or TRAF6 were not due to co-expression we included the human BBK KLHL12 as a further negative control. C2 and F3 inhibited the NF-κB signalling pathway in response to all four stimuli (Fig. 1a-d). To dissect which domain/s of C2 and F3 were needed for this activity, HEK-293T cell lines engineered to express the indicated proteins inducibly were utilised and NF-κB reporter gene assays were repeated. Although both the N-terminal BTB-BACK domains (C2-B) and the C-terminal Kelch domain (C2-K) of C2 each had inhibitory activity, the inhibition was stronger for C2-K (Fig. 1e). Similarly, the Kelch domain of F3 (F3-K) alone inhibited NF-κB reporter gene activation, however the BTB domain of F3 (F3-B) alone was insufficient to inhibit activity (Fig. 1f). Immunoblotting for the Flag tag showed that these proteins were expressed at similar levels (Fig. 1g and h). These findings are consistent with our previous report that the Kelch domain of A55 is required for its inhibition of NF-κB signalling [13]. To obtain independent evidence of NF-κB inhibitory activity, HEK-293T cell lines that inducibly express VACV proteins C6, B14, C2 or F3 were treated with doxycycline to induce protein expression, then stimulated with TNFα and the levels of *IL-8* mRNA was determined by RT-qPCR (Fig. 2a) or the levels of secreted IL-8 were measured by ELISA (Fig. 2b). B14, F3 or C2 each had inhibitory activity relative to VACV protein C6, which inhibits IRF3 activation [47] and JAK-STAT signalling [8, 50] but not NF-κB. To investigate if inhibition of signalling was specific to NF-κB or general, the effect of C2 on expression from the type I IFN-stimulated response element (ISRE) promoter in response to IFN-α was analysed by reporter gene assay. This showed C2 did not inhibit this pathway, whereas C6 did, highlighting its specificity (Fig. S1).

### C2 and F3 inhibit NF-κB at or downstream of p65

**Fig. 3:**
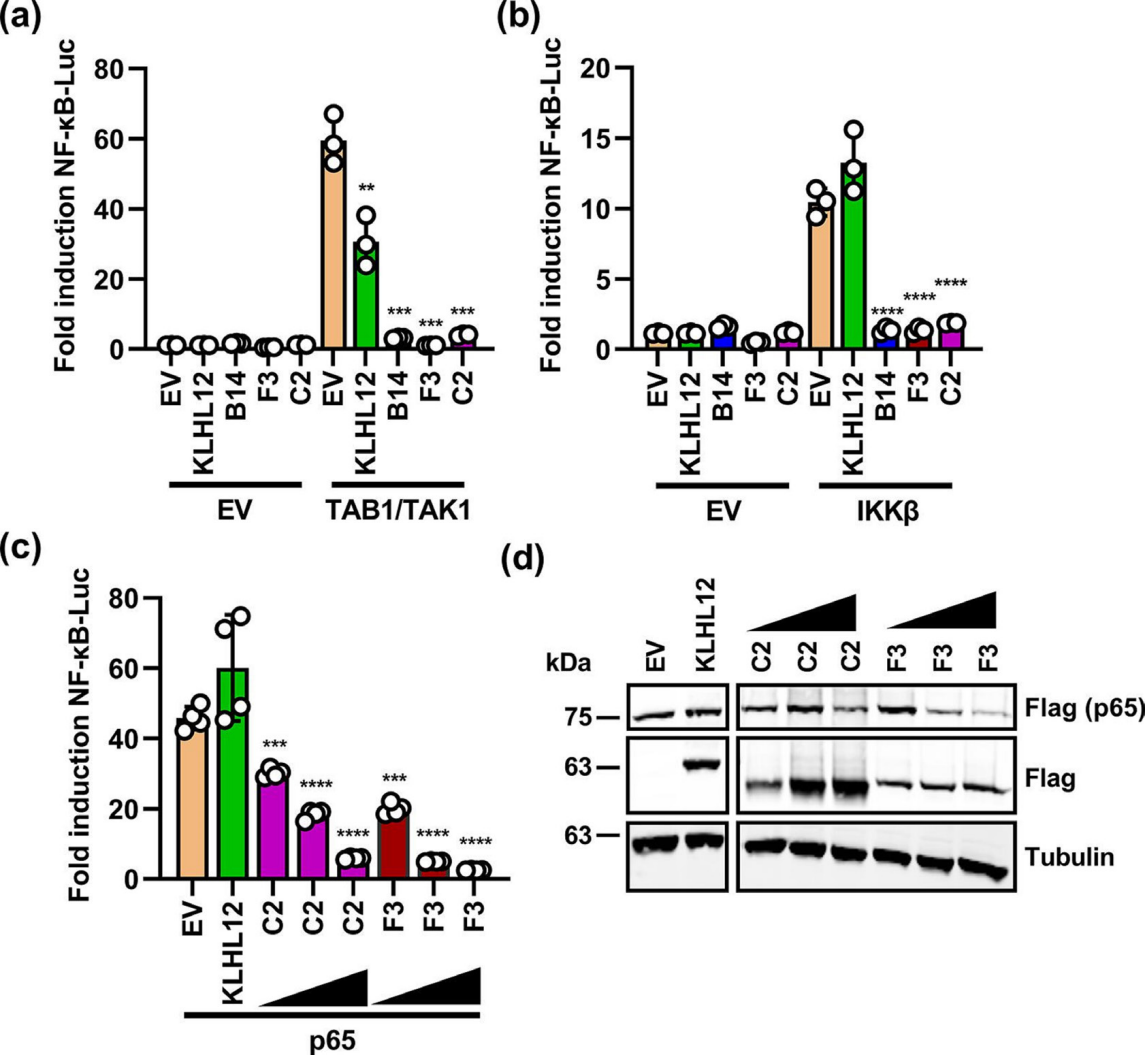
C2 and F3 inhibit NF-κB signalling downstream of p65. (a-c) HEK-293T cells were co-transfected with the pNF-KB-Luc, pTK-RL and 25, 50 or 150 ng of plasmids encoding the indicated TAP-tagged proteins. Cells were untreated or stimulated by co-transfecting plasmids expressing TAK1/TAB1, *IKKβ* or p65. Cells were lysed 24 h after transfection and luminescence was measured as in Fig. 1. (d) Immunoblot indicating protein expression of cells treated in (c); molecular masses in kDa are indicated on the left. Data shown (mean±SD) are representative of three experiments. Statistical analysis was performed by unpaired Student’s t-test (**P <0.01, ***P <0.001, ****P <0.0001) in comparison to stimulated EV control.

To determine at what stage C2 and F3 inhibit the NF-κB pathway, the pathway was activated by overexpression of TAK1/TAB1, IKKβ or p65. Like B14, C2 and F3 inhibited the pathway when it was activated by overexpression of either TAK1/TAB1 or IKKβ (Fig. 3a, b). C2 and F3 also inhibited the pathway in a dose-dependent manner when it was activated by p65 overexpression (Fig. 3c), and both C2 and F3 shared this property with A55, which interacts with KPNA2 and diminishes translocation of p65 into the nucleus [13]. In contrast, the cellular BTB-Kelch protein KLHL12 was generally non-inhibitory. Immunoblotting, showed higher expression of C2 than F3 and equivalent loading was shown by blotting for α-tubulin (lower panels). Notably, F3 expression reduced expression of p65 in a dose-dependent manner (Fig. 3d). Whether this is relevant to the mechanism by which F3 inhibits NF-κB activation or an artificial effect due to promoter competition is unknown, but C2 did not induce reduction in p65 level, despite higher levels of expression. These data suggest C2 and F3 interfere with NF-κB activation at or downstream of p65.

### C2 and F3 do not prevent IκBα degradation

**Fig. 4:**
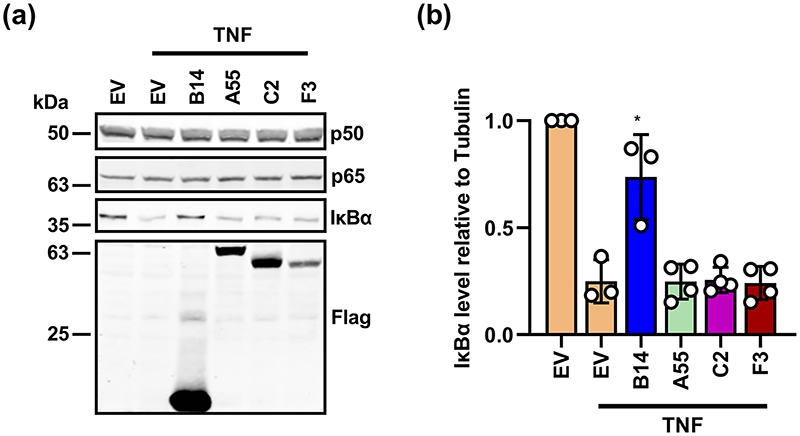
C2 and F3 inhibit NF-κB activation downstream of IκBα degradation. (a) HEK-293T cell lines were induced to express TAP-tagged proteins B14, A55, C2 or F3 or containing empty (EV) by addition of 2 μg ml^−1^ doxycycline for 24 h. Cells were then stimulated with TNFα 20 ng ml^−1^ for 30 min and cell lysates were analysed by immunobotting using antibodies against the proteins indicated on the right. Molecular masses (in kDa) are indicated on the left. (b) Levels of IκBα relative to p50 as quantified by densitometry. Data shown (mean±SD) are an average of three independent experiments and were analysed by an unpaired Student’s t-test (**P <0.01, ***P <0.001, ****P <0.0001) in comparison to stimulated EV control.

The above data were derived from Luciferase based reporter gene assays, RT-qPCR of endogenous genes and ELISA. To have an alternative assessment of NF-κB activation, the level of the inhibitor of κBα (IκBα) was measured by immunoblotting. Upon pathway activation, IκBα is phosphorylated by IKKβ, leading to its ubiquitylation and degradation by the proteasome, which releases the NF-κB subunits p50 and p65 to translocate into the nucleus. To examine if C2 or F3 affect IκBα levels, HEK-293T cell lines inducibly expressing B14, C2, F3 or EV as a control, were stimulated with TNFα for 30 min, and the level of IκBα was examined by immunoblotting. Upon stimulation, in the presence of EV, IκBα was degraded compared to the unstimulated EV cells, whereas it was stabilised by B14, which binds to IKKβ and inhibits IκBα phosphorylation [14]. In comparison, in the presence C2 or F3, IκBα showed normal degradation upon pathway stimulation (Fig. 4a, b). The levels of IκBα relative to an internal control p50 were quantified by densitometry from three experiments and C2 and F3 induced no significant change compared to EV (Fig. 4b). This indicates C2 and F3 act downstream to IκBα degradation, consistent with conclusions from the reporter gene assays.

### C2 and F3 diminish p65 nuclear translocation

**Fig. 5:**
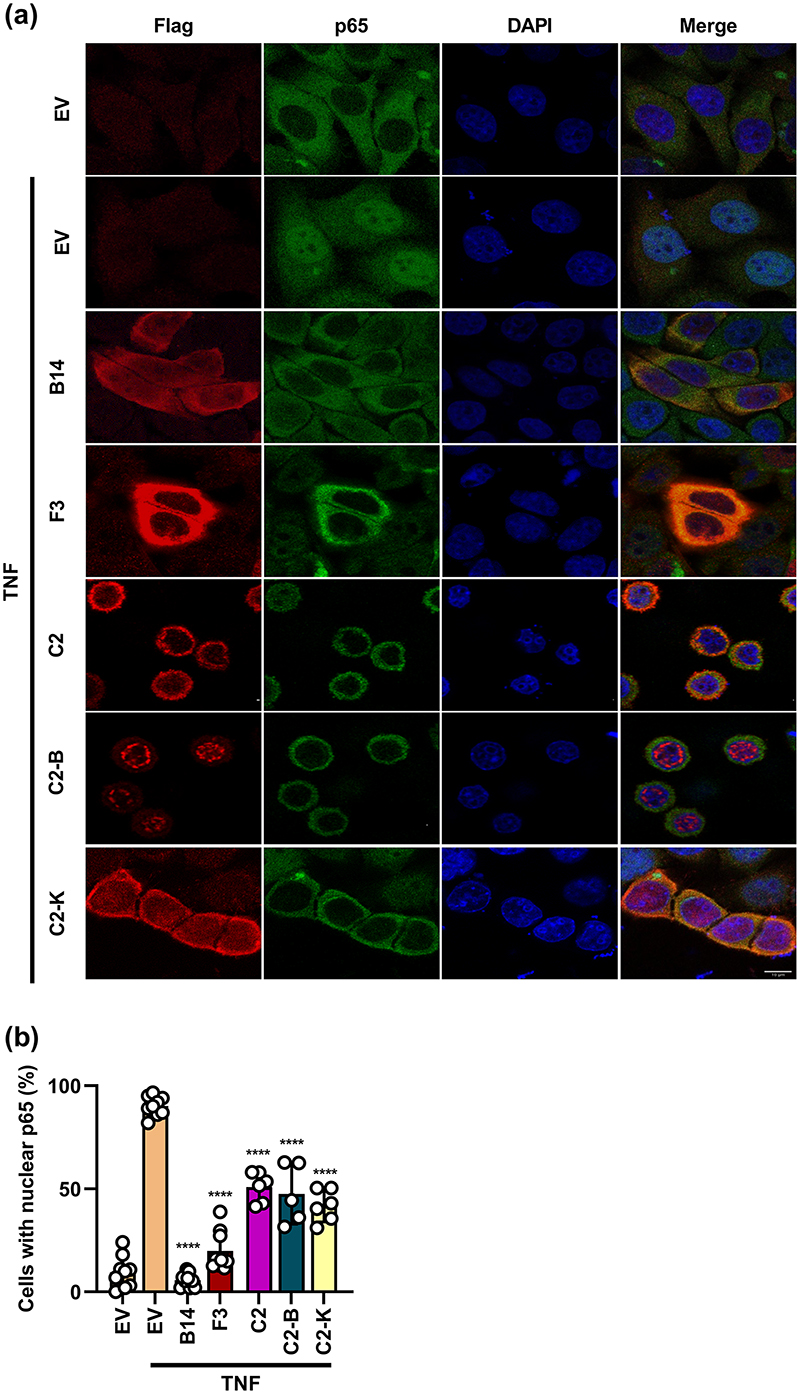
p65 translocation into the nucleus is inhibited by C2 and F3. (a) Immunofluorescent staining of p65 sub-cellular localisation. HeLa cells were transfected with plasmids encoding Flag-tagged B14, F3, C2, C2-B, C2-K or empty vector (EV) for 24 h and then left untreated or stimulated with 40ngml^-1^ TNFα for 30 min. Cells were stained with DAPI (blue), anti-Flag (red) and anti-p65 (green). (b) Average number of transfected cells with nuclear p65. Data shown (mean±SD) are representative of three experiments, each carried out in triplicate and with 100 cells counted per condition. Data were analysed by an unpaired Student’s t-test (****P <0.0001) in comparison to stimulated EV control. The size bar=20 μM.

Next the ability of C2, C2-B, C2-K or F3 to prevent p65 translocation into the nucleus was examined by immunofluorescence. HeLa cells were transfected with plasmids encoding TAP-tagged B14, C2, C2-B, C2-K or empty vector (EV). To exclude p65 from the nucleus prior to stimulation, transfected cells were starved for 3 h in serum-free DMEM prior to stimulation with TNFα for 30 min (Fig. 5a). The proportion of cells with nuclear p65 was quantified from three separate experiments (Fig. 5b). In the EV group, only 15 % of cells showed nuclear p65 in untreated cells, but this increased to 90 % after TNFα stimulation (Fig. 5a, b). In contrast, in the presence of B14, 90 % of p65 remained cytoplasmic after stimulation, as expected. Interestingly, cells expressing C2, C2-B and C2-K (~40-50 %) and F3 (~20 %) all showed diminished p65 nuclear translocation, compared to EV controls (Fig. 5a, b). A complication of these assays was that C2 and C2-B induced changes to cellular architecture including cell rounding (Fig. 5a). Changes in p65 localisation might partly reflect these morphological changes. Importantly, C2-K did not induce these changes and so there is greater confidence that the C2 Kelch domains (aa 213−512) were preventing p65 translocation. Collectively, these data indicate that C2 and F3 inhibit the NF-κB signalling pathway downstream of IκBα degradation and at or upstream of p65 translocation into the nucleus.

### Over-expressed C2 does not co-precipitate with Cul3 or KPNA2

**Fig. 6:**
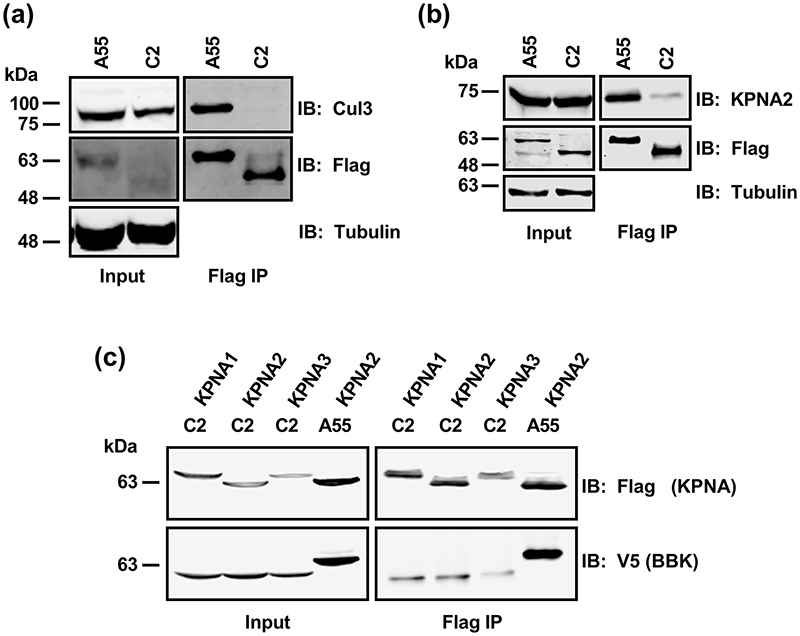
C2 does not co-precipitate with cullin-3 or KPNA2. (a-b) HEK-293T cells were transfected with pcDNA4/TO-nTAP-A55 or pcDNA4/TO-nTAP-C2 for 18 h. Cells were lysed in (a) NP-40/PBS buffer or (b) RIPA buffer followed by Flag-tagged immunoprecipitation (IP) of cleared cell lysates. Co-IP of endogenous (a) cullin-3 (Cul3) or (b) KPNA2 by A55 or C2 was probed with an anti-Cul3 or anti-KPNA2 antibody. (c) HEK-293T cells were co-transfected with pcDNA4/TO-V5-A55 or pcDNA4/TO-V5-C2 with plasmids encoding Flag-tagged KPNA1, KPNA2 or KPNA3. Cells were lysed in RIPA buffer followed by Flag-tagged IP of cleared lysates. Co-IP of A55 or C2 by KPNA1, 2 or 3 was probed with an anti-V5 antibody. Molecular masses (in kDa) are indicated on the left. Immunoblots shown are representative of three independent experiments.

BTB proteins can function as substrate-specific adaptors for protein ubiquitylation in Cul3-based E3 ubiquitin-ligase complexes. The BTB domain interacts with Cul3, whereas the Kelch domain is a substrate-recognition module, leading to the degradation or modification of the substrate [39]. Therefore, to further our understanding of mechanism a possible interaction with Cul3 was investigated. From here on we focused on protein C2 because the relatively poor expression of F3 made protein interaction analyses difficult. Flag-tagged A55 or C2 were expressed by transfection in HEK-293T cells, immunoprecipitated via the Flag-tag and analysed by immunoblotting. This showed that A55 co-precipitated with endogenous Cul3 as reported [13, 44], but C2 did not (Fig. 6a). The failure of C2 to co-precipitate with Cul3 is consistent with the recent structure of the Cul3-A55 BTB-BACK complex in which it was shown that a region of A55 important for Cul3 interaction is missing in C2 [44]. Given that ectopic expression of C2 is sufficient to inhibit NF-κB activation, C2 does not bind Cul3, and the C2-K domain has inhibitory activity, C2 inhibits NF-κB activation in a Cul3-independent mechanism.

VACV BTB-Kelch protein A55 is proposed to restrict p65 nuclear translocation through its interaction with the importin KPNA-2 and disruption of KPNA-2/p65 complex formation [13]. To determine if C2 could also inhibit p65 translocation in this way, the interaction of C2 with importins was studied by immunoprecipitation. Whilst A55 immunoprecipitated endogenous KPNA-2 specifically, as expected, only very weak interaction with C2 was observed despite higher level of expression (Fig. 6b). Consistent with this finding, compared to an observed strong interaction of A55 with KPNA2, only a very weak interaction with C2 was observed following reverse co-IP using Flag-tagged KPNA1, KPNA2 or KPNA3 and V5-tagged A55 or C2 (Fig. 6c). This suggests that the mechanism of inhibition for C2 differs to that of A55.

### Immunisation with a VACV lacking C2 induced stronger CD8+ T cell responses and better protects against challenge

**Fig. 7:**
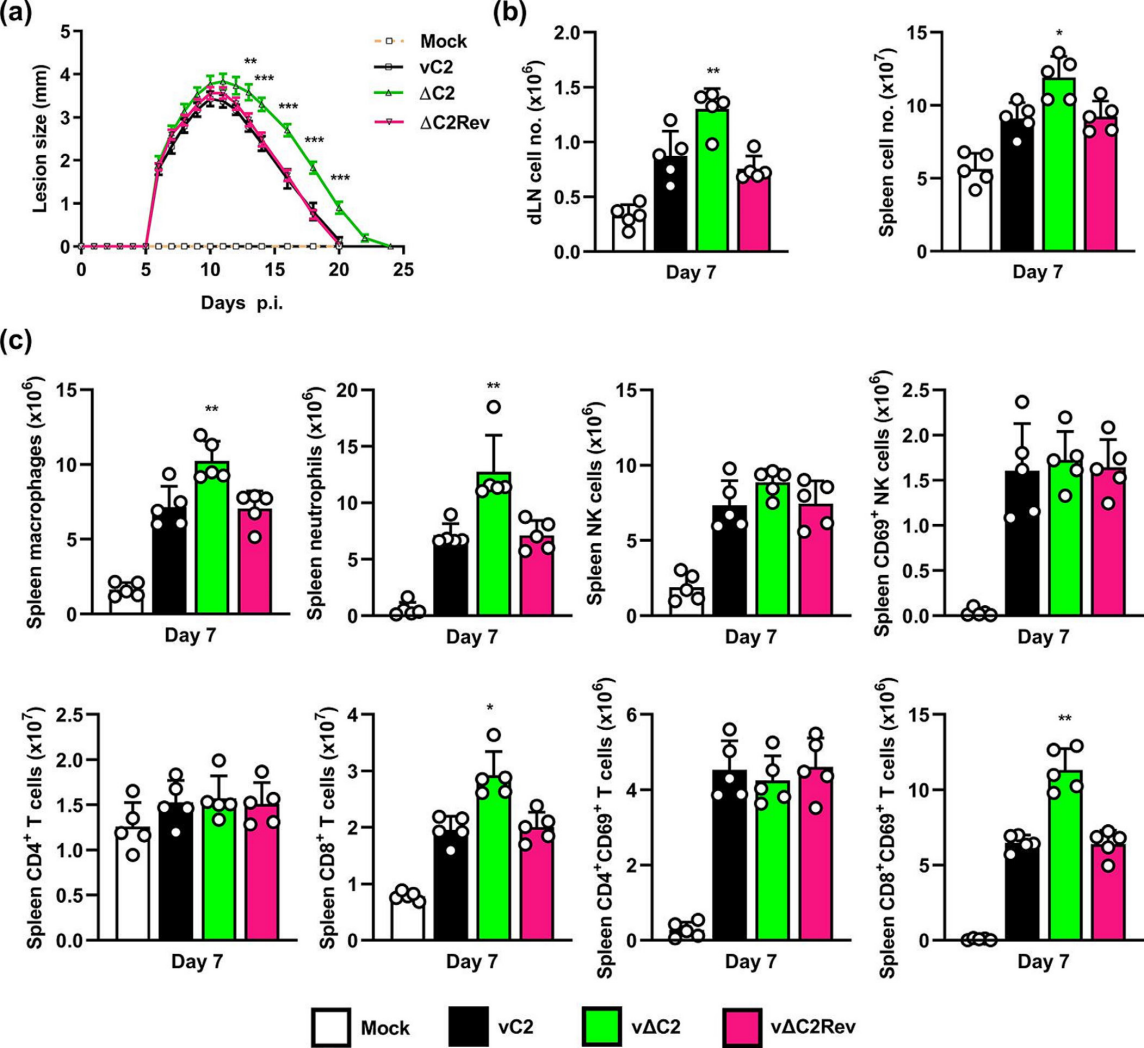
Intradermal infection of mice with vΔC2 induces enhanced acute immune response. C57BL/6 mice (*n*=5) were infected intradermally in the ear pinnae with 1<10^4^ p.f.u. of VACV strains vC2, vΔC2 or vC2Rev or were mock infected with PBS. (a) Lesion sizes were measured daily. Data shown (mean±SD) are from one of two independent experiments and were analysed by an unpaired Student’s t-test (*P <0.05, **P <0.01) comparing vΔC2 to vC2 and vC2Rev. (b) Total cell numbers from the spleen or draining lymph nodes at 7 d p.i. (c) FACs analysis of the absolute number of splenic macrophages, neutrophils, NK cells and CD8^+^ or CD4^+^T cells at 7 d p.i. The number of NK, CD4^+^ or CD8^+^T cells expressing CD69 is also shown. Data shown (mean±SD) are from one of two independent experiments and were analysed by an unpaired Student’s t-test (*P <0.05, **P <0.01) in comparison vΔC2 to vC2 and vC2Rev.

Previously, it was shown that VACV strain WR lacking the *C2L* gene induced altered responses to intradermal infection compared to *C2L* positive viruses [31]. This was repeated here and again showed an increased lesion size after infection by vΔC2 (Fig. 7a). To examine the basis for this difference and determine if infection by vΔC2 induced an alteration in the immune response, mice were infected intradermally with vC2, vΔC2 or vC2-rev and the recruitment of immune cells to the draining lymph node (DLN) and spleen was analysed at 7 d p.i. (Fig. 7b, c). This showed increased recruitment of cells in both spleen and DLN by vΔC2 (Fig. 7b). Next the type of cells in the spleen and their activation status was examined by flow cytometry (Fig. 7c). No differences were seen in NK cells or CD4+ T cells, when comparing vΔC2 with control viruses, however, the number of macrophages, neutrophils and CD8+ T cells were increased following infection by vΔC2. Further, the CD8+ T cells showed greater activation as judged by CD69 expression (Fig. 7c).

**Fig. 8:**
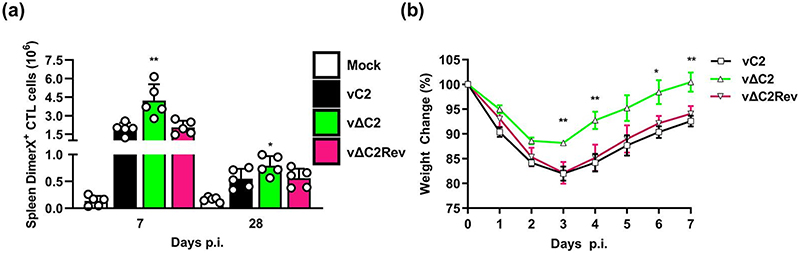
Infection with vΔC2 provides enhanced CD8^+^ Tcell response and enhanced protection against challenge. Mice were infected as in Fig. 7. (a) At 7 or 28 d p.i. the number of splenic DimerX positive CTL cells were determined by FACs. Data shown (mean±SD) are from one of two independent experiments and were analysed by an unpaired Student’s t-test (*P <0.05, **P <0.01) comparing vΔC2 to vC2 and vC2Rev. (b) Weight loss following intranasal infection of mice immunized with vC2, vΔC2 or vC2Rev 28 d earlier. Data shown (mean±SEM) of one of two independent experiments and were analysed by an unpaired Student’s t-test (*P <0.05, **P <0.01) in comparison vΔC2 to vC2 and vC2Rev.

Given enhanced numbers and enhanced activation of CD8^+^ T cells, the proportion of VACV-specific, splenic, CD8^+^ T cells was examined by tetramer staining at 7 and 28 d p.i. (Fig. 8a). At both time points there were increased numbers of VACV-specific CD8^+^ T cells after infection with vΔC2 compared to controls. The enhanced CD8^+^ T cell memory response, suggested that there might be altered protection against re-infection with VACV. This was addressed by infecting immunised mice intranasally with wild-type VACV at 28 d p.i. and measuring weight change over the next 7 d. Notably, animals immunised with vΔC2 showed reduced weight loss after challenge compared to mice immunised with control viruses (Fig. 8b). Therefore, loss of the *C2L* gene from VACV WR has improved the immunogenicity of the virus.

## Discussion

NF-κB is an important transcription factor that is activated during the response to infection by pathogens and promotes the innate immune response. Accordingly, many pathogens target it and modulate its activity. This is well illustrated by VACV, which, prior to this study, was known to express twelve different intracellular inhibitors of this pathway. Here, two additional VACV-encoded NF-κB inhibitors are described and characterised, protein C2 and F3. Both proteins affect the outcome of infection *in vivo* despite the presence of other NF-κB inhibitors [31, 33], showing that these proteins have non−redundant functions.

Like the other NF-κB inhibitors, protein C2 and F3 are expressed early during infection and are non-essential for virus replication, but function within the cytosol to block the pathway downstream of receptors for TNFα and IL- 1β. Mapping the site of inhibition showed that C2 and F3 act quite late in the pathway to reduce p65 translocation into the nucleus and F3 also reduced p65 expression levels. Therefore, these proteins act at a similar site in the pathway to A55, the other VACV BTB-Kelch protein. Exactly how C2 and F3 prevent p65 translocation remains to be determined. Collectively, these three BTB-Kelch proteins act to diminish p65 translocation into the nucleus and represent the penultimate VACV-mediated blockade of the pathway. The only downstream inhibitor is protein F14 that functions within the nucleus to block the interaction between p65 and histone acetylase CBP by molecular mimicry [30].

Host BTB-Kelch proteins often interact with Cul3 via their BTB-BACK domains, and with specific substrates via their C-terminal Kelch domain. In this way, substrates are brought to the E3-ubiquitin ligase complex and ubiquitylated to induce proteasomal degradation or modification of function. Recently, VACV protein A55 was shown to inhibit NF-κB activation and to co-precipitate with Cul3 [13]. This interaction was direct and the co-crystal structure of the A55 BTB-BACK domain in complex with the N-terminal domain of Cul3 was determined [44]. Biophysical measurement showed A55 had a much higher affinity for Cul3 than did cellular BTB proteins, suggesting that A55 would be an effective competitor for Cul3 binding [44]. Despite this, the interaction with Cul3 was not the mechanism by which A55 inhibited NF-κB because the Kelch domain was sufficient to inhibit the pathway and did this by binding to KPNA2 and thereby preventing translocation of p65 into the nucleus [13]. In comparison, C2 inhibits the pathway at a similar stage and also does this in a Cul3-independent way, because C2 does not bind Cul3. However, an interaction between C2 and KPNA2 was not observed, suggesting that the mechanism of inhibition by C2 and A55 differ and for C2 remains to be determined.

The outcome of infection with a virus lacking C2 was compared with viruses expressing C2. Previously, infection with vΔC2 induced larger dermal lesions with an increased infiltration of neutrophils, T cells and macrophages [31]. Here, infection with vΔC2 also induced larger lesions, and there were also greater numbers of cells in the DLN and spleen at 7 d p.i. These cells included greater numbers of macrophages, neutrophils and CD8^+^ T cells, and the CD8^+^ T cells showed enhanced activation (CD69+). In addition, the VACV-specific, CD8^+^ T cell response was enhanced at both day 7 and 28. The stronger VACV-specific, splenic, memory, T cell response induced by vΔC2 correlated with reduced weight loss following intranasal change on day 28, showing vΔC2 is a more potent vaccine. Although, it is not proven that the enhanced CD8^+^ T cell response is the cause of enhanced protection, enhanced CD8^+^ T cell responses were induced by other VACV strains from which genes encoding NF-κB inhibitors had been deleted, consistent with the notion that NF-κB activation has an important role in the development of CD8^+^ T cell immunological memory. Examples include the BTB-Kelch protein A55 [13], and the Bcl-2 proteins N1 [49], A46 [51] and K7 [52], although deletion of immunomodulators of other pathways, such as C6, can also affect CD8^+^ T cell memory C6 [53, 54], for review see [55]. When considering recombinant vaccine design it is notable that although the deletion of single immunomodulators enhanced immunogenicity, deletion of multiple immunomodulators decreased immunogenicity [56].

Overall, this study identifies VACV encoded F3 and C2 as immunomodulators and additional inhibitors of the NF-κB signalling and establishes that, despite the presence of multiple NF-κB inhibitors, immunisation with vΔC2 enhances CD8^+^ T cell memory and protection from re-infection.

## Supplementary Material

Supplementary material
