# Supplementary material for: Vaccinia virus BTB-Kelch proteins C2 and F3 inhibit NF-κB activation
Source: J Gen Virol. Author manuscript; Available in PMC 2023 Jul 31. (PMC7614845; doi:10.1099/jgv.0.001786)
Supplement: Supplementary material [file EMS179320-supplement-Supplementary_material.pdf]

Construct	Insert	Vector	Ref. /source
nTAP-A55 *	TAP- codon optimised (co) A55	pcDNA4/TO (Invitrogen)	Smith lab
nV5-A55	V5- co A55	pcDNA3 (Invitrogen)	Smith lab
nTAP-C2	TAP- co C2	pcDNA4/TO (Invitrogen)	Smith lab
nV5-C2	V5- co C2	pcDNA3 (Invitrogen)	Smith lab
nV5-GFP	V5-GFP	pcDNA3 (Invitrogen)	Smith lab
nTAP-F3	TAP- co F3	pcDNA4/TO (Invitrogen)	Smith lab
nTAP-F3 BTB	TAP- co F3 BTB BACK domain	pcDNA4/TO (Invitrogen)	Smith lab
nTAP-F3 Kelch	TAP- co F3 Kelch	pcDNA4/TO (Invitrogen)	Smith lab
nTAP-C2-BTB	TAP- co C2 BTB BACK domain	pcDNA4/TO (Invitrogen)	Smith lab
nTAP-C2-Kelch	TAP- co C2 Kelch domain	pcDNA4/TO (Invitrogen)	Smith lab
cTAP-B14	Co B14-TAP	pcDNA4/TO (Invitrogen)	Smith lab
Flag-KLHL12	Flag-Kelch like protein 12	pcDNA3 (Invitrogen)	Gift from Randall T. Moon, University of Washington
Flag-TRAF6	Flag-TRAF6	M5P	Gift from Andrew Bowie, Trinity College Dublin
Flag-TRAF2	Flag-TRAF2	M5P	Gift from Andrew Bowie lab, Trinity College Dublin
pcDNA3-TAK1	HA-TAK1	pcDNA3 (Invitrogen)	Smith lab
pcDNA3-TAB1	HA-TAB1	pcDNA3 (Invitrogen)	Smith lab
pLuc-NF-κB	NF-κB promoter fused to a firefly luciferase gene		A gift from R. Hofmeister (University of Regensburg)
pLuc-ISRE	IFN-stimulated response element fused to luciferase gene	pLuc-ISRE	Promega
pRL-SV40P	Renilla SV40 promoter (no enhancer) reporter gene	pRL-TK	A gift from Ron Prywes (Addgene plasmid # 27163)
pCMV-PACK	Packaging plasmid for lentivirus production with HIV Gag, Pol, Rev and Tat under the CMV promoter; Amp-R	pCMV	Gift from H. Laman
pCMV-ENV	VSV-G pseudotyped envelop protein under the CMV promoter for lentivirus production; Amp-R	pCMV	Gift from H. Laman

pLDT-TetR	Tetracycline promoter repressor	pLKO puro	1
pLDT-nTAP-A55	TAP- co A55	pLKO puro	1
pLDT-nTAP-F3	TAP- co F3	pLKO puro	Smith lab
pLDT-nTAP-C2	TAP- co C2	pLKO puro	Smith lab
pLDT-nTAP-C2-BTB	TAP- co C2 BTB BACK	pLKO puro	Smith lab
pLDT-nTAP-C2-Kelch	TAP- co C2 Kelch	pLKO puro	Smith lab
pLDT-nTAP-B14	TAP-co B14	pLKO puro	Smith lab
pLDT-nTAP-C6	TAP- co C6	pLKO puro	Smith lab

Table S1. Plasmids used in this study. *TAP, short for tandem affinity purification, contains two Streptavidin binding sequences and one Flag tag.

References

1. Everett RD, Bell AJ, Lu Y, Orr A. 2013. The replication defect of ICP0-null mutant herpes simplex virus 1 can be largely complemented by the combined activities of human cytomegalovirus proteins IE1 and pp71. J Virol **87**:978–990. doi:10.1128/JVI.01103-12.

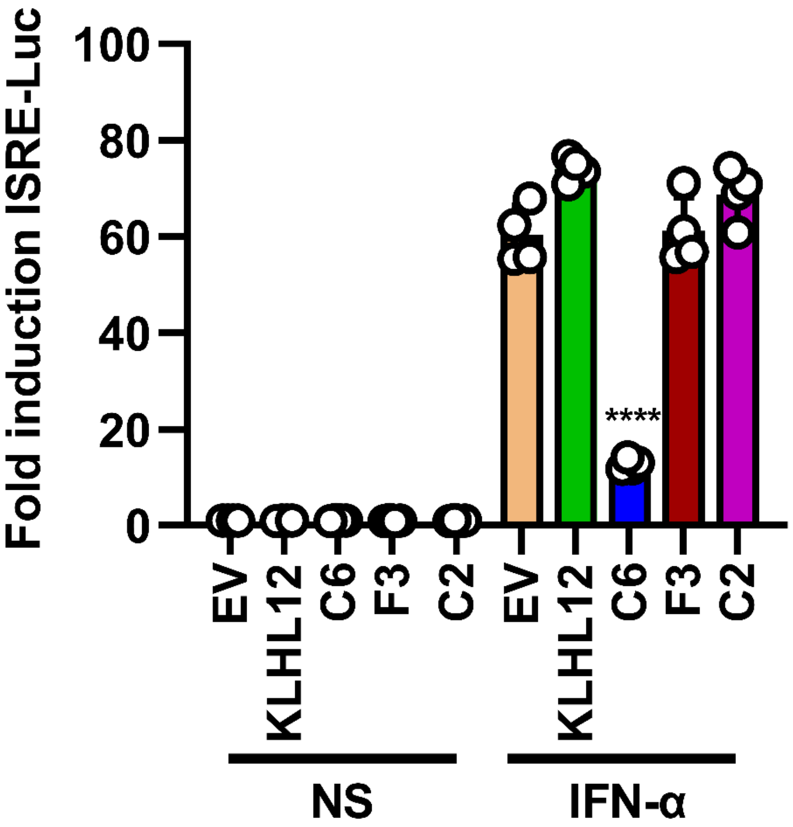

Fig. S1. C2 and F3 do not inhibit IFN-α-dependent signalling. HEK-293T cells were co-transfected with pISRE-Luc, pTK-RL and plasmids for expression of the indicated TAP-tagged proteins. Eighteen hours post-transfection cells were untreated or treated by addition of IFN-α (1000 U/ml) for 6 h. Luciferase activity was measured in cell lysates and data are expressed as the fold change in Firefly luciferase relative to Renilla and EV unstimulated. Data shown (mean ± SD) are representative of 3 experiments and were analysed by an unpaired Student's t-test (** P<0.01, *** P<0.001, **** P<0.0001) in comparison to stimulated EV.
